# Editorial: Mechanisms of endocrine cell proliferation in the embryonic, neonatal and adult pancreas

**DOI:** 10.3389/fendo.2026.1917333

**Published:** 2026-07-03

**Authors:** Esra Karakose, Erick Spears, Ioannis Serafimidis

**Affiliations:** 1 Diabetes, Obesity and Metabolism Institute, Icahn School of Medicine at Mount Sinai, New York, NY, United States; 2 Department of Biology, Belmont University, Nashville, TN, United States; 3 Center of Basic Research, Biomedical Research Foundation of the Academy of Athens, Athens, Greece

**Keywords:** diabetes, endocrine cell plasticity, endocrine cell proliferation, pancreatic islets, β-cell regeneration

The maintenance of glucose homeostasis depends on a sufficient population of hormone-producing endocrine cells within the pancreatic islets, particularly insulin-secreting β-cells. Loss of β-cell mass and function is a defining feature of both type 1 and type 2 diabetes (1), making the restoration or expansion of functional endocrine cell populations a major goal of regenerative medicine (2, 3). In this context, understanding the mechanisms that govern endocrine cell proliferation throughout embryonic, neonatal, and adult life remains of fundamental biological and clinical importance.

Endocrine cell proliferative capacity changes dramatically across the lifespan. During embryonic and neonatal development, endocrine cells undergo extensive expansion (4), whereas adult endocrine cells are largely quiescent under physiological conditions (5). Nevertheless, proliferation can be reactivated in response to metabolic stress, injury, or therapeutic intervention (6, 7), highlighting the existence of latent regenerative mechanisms. Identifying the signals that enable or constrain endocrine cell proliferation at different stages of life is therefore essential for the development of regenerative strategies for diabetes (8). The contributions assembled in this Research Topic address this challenge through studies of developmental programming, epigenetic regulation, nutrient sensing, metabolic adaptation, and microenvironmental control of endocrine cell growth, collectively advancing our understanding of endocrine cell proliferation and regeneration across the lifespan.

A major theme emerging from this Research Topic is the regulation of endocrine cell proliferation and the maintenance of endocrine cell mass. Although the underlying mechanisms are diverse, several contributions converge on the concept that endocrine cells must balance proliferative capacity, survival, and functional maturation in order to meet changing physiological demands. A notable example is the study by Zhang et al. on β-cell-specific deletion of Pdgfrα. While the authors demonstrate impaired glucose homeostasis and increased β-cell apoptosis, they also report a marked reduction in proliferating Ki67+ β-cells within mutant islets, highlighting an essential role for PDGFRα signaling in maintaining β-cell proliferative competence. These findings extend previous studies implicating PDGF signaling in postnatal β-cell expansion (9) and identify the PI3K-Atf5 pathway as a potential molecular link between growth factor signaling and endocrine cell mass maintenance.

Similarly, the study by Chiba et al. investigating PFKFB3 demonstrates how metabolic adaptation may support endocrine cell expansion under conditions of increased demand. PFKFB3 expression was induced in response to acute metabolic stress both *in vitro* and *in vivo*, and disruption of this pathway impaired insulin secretion, glucose tolerance, and cellular metabolic activity. These findings suggest that metabolic remodeling is not merely a consequence of endocrine cell activation but a permissive mechanism supporting adaptation and compensatory growth.

A second major theme concerns the importance of developmental and epigenetic mechanisms in establishing endocrine cell identity and proliferative potential. The review by Deng and Krishnamurthy highlights the growing utility of patient-derived induced pluripotent stem cells (iPSCs) for modeling monogenic disorders of β-cell function, including maturity-onset diabetes of the young and congenital hyperinsulinism. Beyond disease modeling, these systems provide powerful platforms for investigating how disease-causing variants influence endocrine lineage specification, β-cell maturation, and endocrine cell generation. Complementing this work, Arroyave et al. identified the histone demethylase KDM4A as an essential epigenetic regulator of β-like cell differentiation. Suppression of KDM4A impaired key pancreatic transcription factors and reduced glucose-stimulated insulin secretion, highlighting the importance of chromatin remodeling in endocrine cell development (10).

Several studies further highlight the close relationship between adaptive endocrine cell growth and diabetes pathogenesis. For example, Odyjewska et al. showed that in children and adolescents with type 1 diabetes, residual C-peptide secretion was associated with elevated circulating betatrophin levels. Although the precise biological role of betatrophin remains controversial, its original identification as a factor linked to β-cell expansion makes this association particularly intriguing. Whether betatrophin contributes directly to preservation of residual β-cell mass or serves as a biomarker of adaptive responses remains unclear.

The relationship between adaptive growth responses and diabetes is also evident in the study of tacrolimus-induced diabetes in obese rats. Teixido-Trujillo et al. demonstrate that tacrolimus administration results in severe hyperglycemia accompanied by reduced β-cell proliferation and altered expression of key β-cell transcription factors. These findings suggest that suppression of compensatory β-cell expansion may represent an important mechanism through which immunosuppressive therapies accelerate diabetes development in metabolically vulnerable individuals.

While islet regeneration research has largely focused on β-cells, increasing evidence suggests that other endocrine cell populations also contribute to islet adaptation and regenerative responses. In particular, α-cells have emerged as important regulators of islet plasticity and may represent a potential source of new β-cells under specific physiological and experimental conditions (11). Accordingly, this Research Topic broadens the discussion to include the α-cell compartment. The study examining the amino acid transporter SLC38A5 (Sellick et al.) identifies a direct, cell-autonomous mechanism linking nutrient availability to α-cell expansion. Genetic deletion of Slc38a5 markedly attenuated amino acid-induced α-cell proliferation despite comparable levels of circulating amino acids, establishing SLC38A5 as a critical component of nutrient-sensing pathways that regulate endocrine cell growth. These findings emphasize that adaptive proliferation is not unique to β-cells but represents a fundamental property of multiple endocrine cell populations within the islet. Subsequent work identified the arginine transporter SLC7A2 as an upstream regulator of amino acid-dependent α-cell proliferation, further highlighting the importance of nutrient sensing in endocrine cell growth (12).

Another important aspect addressed in this Research Topic is the influence of the extracellular microenvironment on endocrine cell function and regenerative potential. The study evaluating extracellular matrix scaffolds derived from decellularized porcine organs (Dhandapani et al.) demonstrates that tissue-derived matrices can provide structural and biochemical support for insulin-secreting cells and isolated pancreatic islets. Several matrices supported endocrine cell attachment, viability, and proliferation while preserving insulin secretory responses. These findings highlight the importance of recreating physiologically relevant microenvironments capable of supporting endocrine cell survival and expansion, an increasingly important consideration for tissue engineering and cell replacement approaches.

Taken together, the contributions assembled in this Research Topic highlight the diverse and interconnected mechanisms that regulate endocrine cell proliferation and endocrine cell mass. Despite addressing diverse biological questions and experimental systems, the studies collectively demonstrate that endocrine cell expansion is governed by developmental, epigenetic, metabolic, and microenvironmental signals (13, 14).

Collectively, these studies reinforce the notion that mechanisms established during development continue to influence endocrine cell plasticity and regenerative potential throughout life (15). By integrating insights from stem-cell models, genetic animal studies, clinical investigations, and tissue-engineering approaches, the articles in this Research Topic advance our understanding of how endocrine cell populations are generated, maintained, expanded, and, under appropriate conditions, regenerated. These findings provide a foundation for future efforts to preserve, restore, or expand functional endocrine cell mass in diabetes.
